# Evaluation of the NMR-MOUSE as a new method for continuous functional monitoring of the small intestine during different perfusion states in a porcine model

**DOI:** 10.1371/journal.pone.0206697

**Published:** 2018-11-02

**Authors:** Paula R. Keschenau, Hanna Klingel, Silke Reuter, Ann Christina Foldenauer, Jochen Vieß, Dennis Weidener, Julia Andruszkow, Bernhard Bluemich, René Tolba, Michael J. Jacobs, Johannes Kalder

**Affiliations:** 1 Department of Vascular Surgery, European Vascular Center Aachen-Maastricht, RWTH University Hospital Aachen, Aachen, Germany; 2 Institut für Technische und Makromolekulare Chemie, RWTH University Aachen, Aachen, Germany; 3 Institute of Medical Statistics, RWTH University Hospital Aachen, Aachen, Germany; 4 Institute for Pathology, RWTH University Hospital Aachen, Aachen, Germany; 5 Institute for Laboratory Animal Science and Experimental Surgery, RWTH University Aachen, Aachen, Germany; 6 Department of Vascular Surgery, European Vascular Center Aachen-Maastricht, AZM University Hospital Maastricht, Maastricht, The Netherlands; Henry Ford Health System, UNITED STATES

## Abstract

**Objective:**

The study aim was to evaluate a small low-field NMR (nuclear magnetic resonance) scanner, the NMR-MOUSE^®^, for detecting changes in intestinal diffusion under different (patho-) physiological perfusion states.

**Methods:**

Laparotomy was performed on 8 female landrace pigs (body weight 70±6 kg) and the feeding vessels of several intestinal loops were dissected. Successively, the intestinal loops were examined using O2C (oxygen to see, LEA Medizintechnik GmbH, Giessen, Germany) for microcirculatory monitoring and the NMR-MOUSE^®^ for diffusion measurement (fast and slow components). On each loop the baseline measurement (physiological perfusion) was followed by one of the following main procedures: method 1 –ischemia; method 2 –flow reduction; method 3 –intraluminal glucose followed by ischemia; method 4 –intraluminal glucose followed by flow reduction. Additionally, standard perioperative monitoring (blood pressure, ECG, blood gas analyses) and histological assessment of intestinal biopsies was performed.

**Results:**

There was no statistical overall time and method effect in the NMR-MOUSE measurement (fast component: p_time_ = 0.6368, p_method_ = 0.9766, slow component: p_time_ = 0.8216, p_method_ = 0.7863). Yet, the fast component of the NMR-MOUSE measurement showed contrary trends during ischemia (increase) versus flow reduction (decrease). The slow-to-fast diffusion ratio shifted slightly towards slow diffusion during flow reduction. The O2C measurement showed a significant decrease of oxygen saturation and microcirculatory blood flow during ischemia and flow reduction (p < .0001). The local microcirculatory blood amount (rHb) showed a significant mucosal increase (p_Clamping(method 1)_ = 0.0007, p_Clamping(method 3)_ = 0.0119), but a serosal decrease (p_Clamping(method 1)_ = 0.0119, p_Clamping(method 3)_ = 0.0078) during ischemia. The histopathological damage was significantly higher with increasing experimental duration and at the end of methods 3 and 4 (p < .0001,Fisher-test).

**Conclusion:**

Monitoring intestinal diffusion changes due to different perfusion states using the NMR-MOUSE is feasible under experimental conditions. Despite the lack of statistical significance, this technique reflects perfusion changes and therefore seems promising for the evaluation of different intestinal perfusion states in the future. Beforehand however, an optimization of this technology, including the optimization of the penetration depth, as well as further validation studies under physiological conditions and including older animals are required.

## Introduction

Intestinal ischemia/reperfusion remains a problem following cardiovascular surgery with extracorporeal circulation (ECC). Although ECC protects from severe visceral ischemia during aortic cross-clamping, it does not prevent moderate intestinal damage [1,2]. Subsequently, the impairment of the intestinal barrier function can lead to serious complications such as a systemic inflammatory response syndrome (SIRS) and multiple organ dysfunction (MOD) [3,4]. In a porcine model, we have been able to identify intestinal microcirculatory dysfunction during ECC due to a perfusion shift between the gut wall layers as the underlying pathophysiological mechanism [5]. However, the exact effects of this microcirculatory dysbalance on the intestinal function are not fully understood. Further investigations are necessary but adequate techniques for non-invasive continuous functional monitoring of the small intestine are lacking.

In other experimental settings, a small low-field NMR (nuclear magnetic resonance) scanner, the so-called NMR-MOUSE (Mobile Universal Surface Explorer), has shown promising results assessing the state of health of biological tissues by means of diffusion measurements [6–8]. The most advanced type of a NMR-MOUSE is the Fourier MOUSE^®^. Depending on zero lateral gradients and a constant vertical gradient along the depth direction it allows to measure depth profiles (relaxation or diffusion) without the time-consuming repositioning of the sensor [7]. A Fourier Transformation (FT) of the data acquired simultaneously for a range of depths (different Larmor frequencies) then leads to a one-dimensional depth-profile with the desired relaxation information.

The key function of the small intestine is absorption of nutrients based on passive and active diffusion as well as on different kinds of transporting processes. But absorption impairment due to hypoperfusion during cardiopulmonary bypass has been reported [9,10]. Therefore, diffusion assessment of the small intestine using the NMR-MOUSE could possibly allow early detection of perfusion changes. The aim of this study was to evaluate diffusion measurement using the NMR-MOUSE as a new method for continuous functional monitoring of the small intestine in an experimental setting of different perfusion states.

## Methods

This study was approved by the Governmental Animal Care and Use Office (Landesamt für Natur, Umwelt und Verbraucherschutz Nordrhein-Westfalen, Recklinghausen, Germany; project/permit number 84-02-04-2013-A447) and was performed in accordance with the German legislation governing animal studies following The Principles of Laboratory Animal Care (NIH publication. 85–23, revised 1996).

8 young female landrace pigs (body weight 70 ± 6 kg) from a disease-free breeding facility (Heinrichs Tierzucht GmbH, Heinsberg, Germany) were chosen for the present study. In order to allow proper acclimatization, the animals were transported at least five days prior to surgery to our local institute where they were housed at 22°C und 55% relative humidity. Before surgery, the animals were fasted over night with free access to water.

### Anesthesia and perioperative monitoring

Intramuscular premedication (0.1 mL/kg ketamine, 0.2 mL/kg azaperone and 1 mL 1% atropine) was followed by intravenous induction of general anesthesia (pentobarbital 10–25 mg/kg) and endotracheal intubation. Anesthesia was maintained using Fentanyl 45 to 90 μg/kg/h i.v. and Isoflurane 1,5–3%. The animals were ventilated using IPPV ventilation with 50% oxygen with a PEEP of 5 mmHg. The perioperative monitoring included invasive measurement of the arterial blood pressure (ABP) and central venous pressure (CVP), ECG monitoring, and measurement of blood oxygen saturation and body temperature.

### Operative procedure

**Fig 1:**
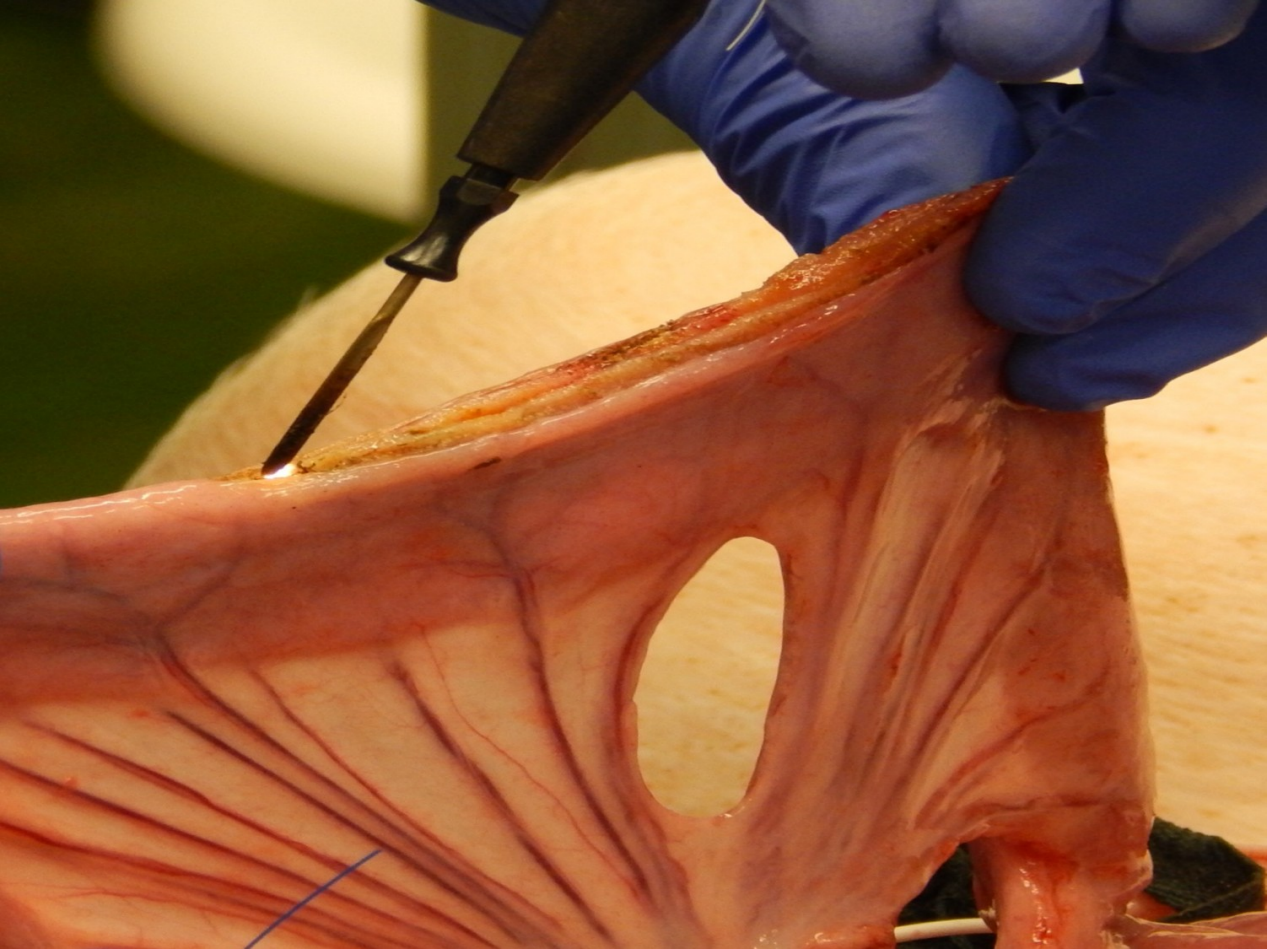
Longitudinal incision of the intestine at the antimesenteric side during the surgical preparation.

**Fig 2:**
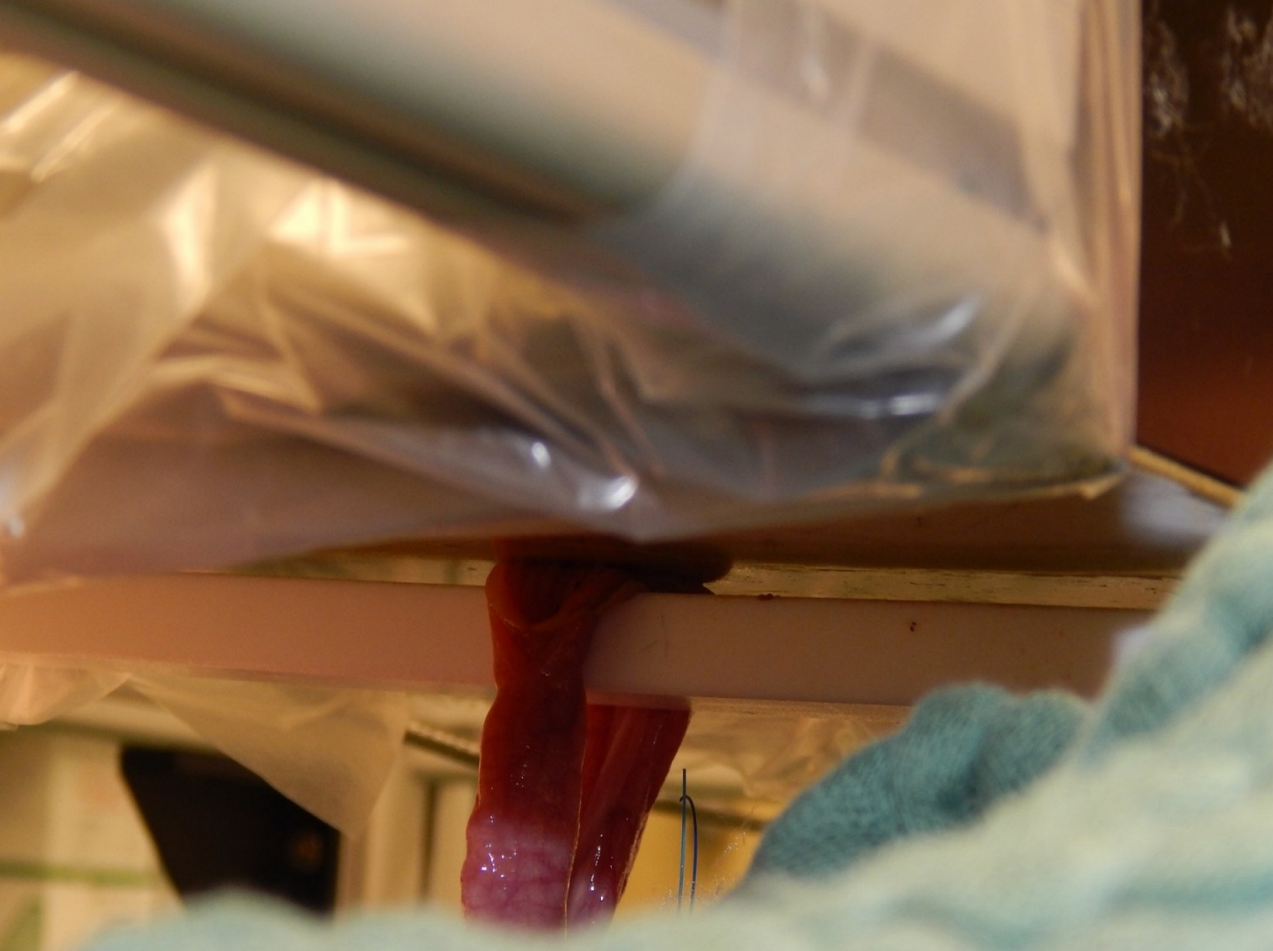
Intraoperative setup for NMR-MOUSE measurement. The intestinal loop has been placed onto the holder of the NMR-MOUSE so that the mucosa touches the sensor lightly.

**Fig 3:**
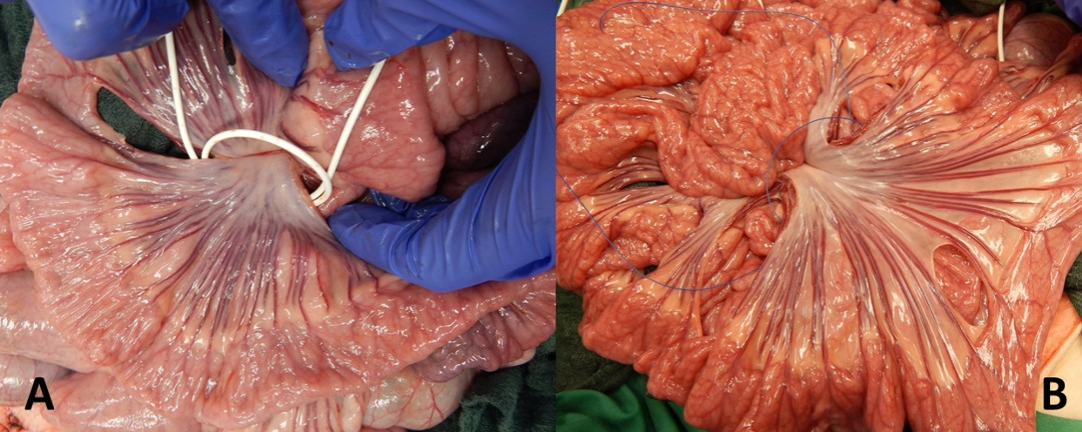
**Intraoperative preparation of an intestinal sling for flow reduction (A) or ischemia (B).** A vessel loop for induction of flow reduction (A) or a Prolene ligature for induction of ischemia (B) have been put in place around the feeding vessels of an intestinal loop during the surgical preparation.

**Fig 4:**
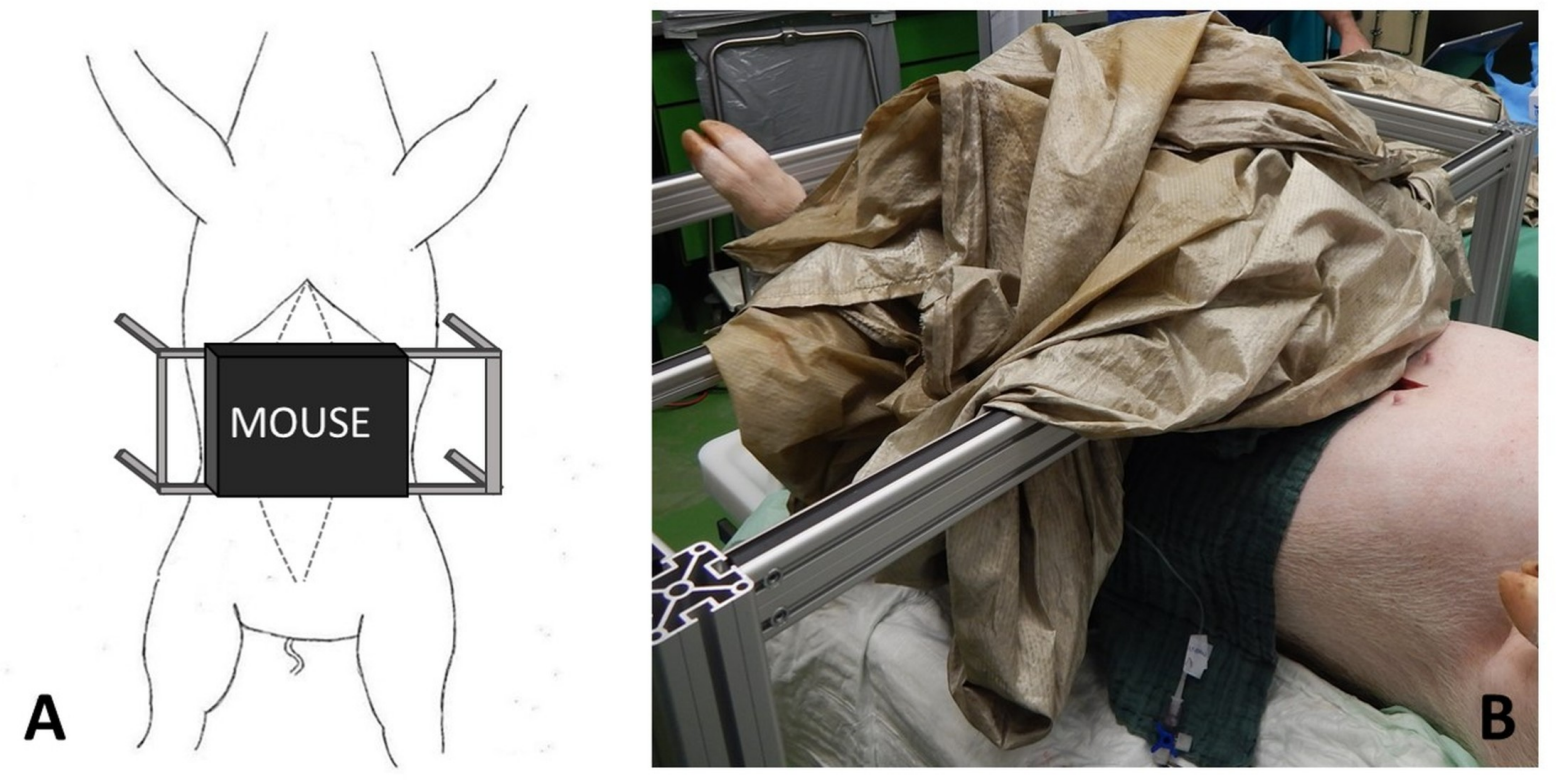
Experimental setup. A: Schematic drawing of the experimental setup with the NMR-MOUSE. B: In the intraoperative setup the NMR-MOUSE was shielded with a granded electrically conducting parachute silk.

After performing a surgical cut down to the carotid artery and the jugular vein and placement of monitoring catheters for measurement of ABP and CVP a median laparotomy was performed. Another catheter was placed into the proximal mesenteric vein for blood sampling during the experiment. The small intestine was exposed and several intestinal loops were marked starting from the proximal jejunum. At least 5 cm distance were kept between the loops in order to minimize possible influence from adjacent intestinal segments. The feeding arteries and veins of each loop were exposed at their mesenteric origin. After the preparation, the loops were examined successively: The intestine was incised longitudinally at the antimesenteric side over a distance of several centimeters (Fig 1). The inner intraluminal and the outer serosal O2C probes were set in place and the loop was placed carefully onto the holder of the NMR-MOUSE, the mucosa touching the MOUSE sensor lightly (Fig 2). Depending on the planned main procedure, a vessel loop for flow reduction or a Prolene ligature for induction of ischemia was prepared loosely around the origin of the feeding vessels of the intestinal loop (Fig 3). With all measuring probes in place and after shielding the MOUSE with a granded electrically conducting parachute silk the measurements were started. Fig 4 shows the experimental setup.

**Fig 5:**
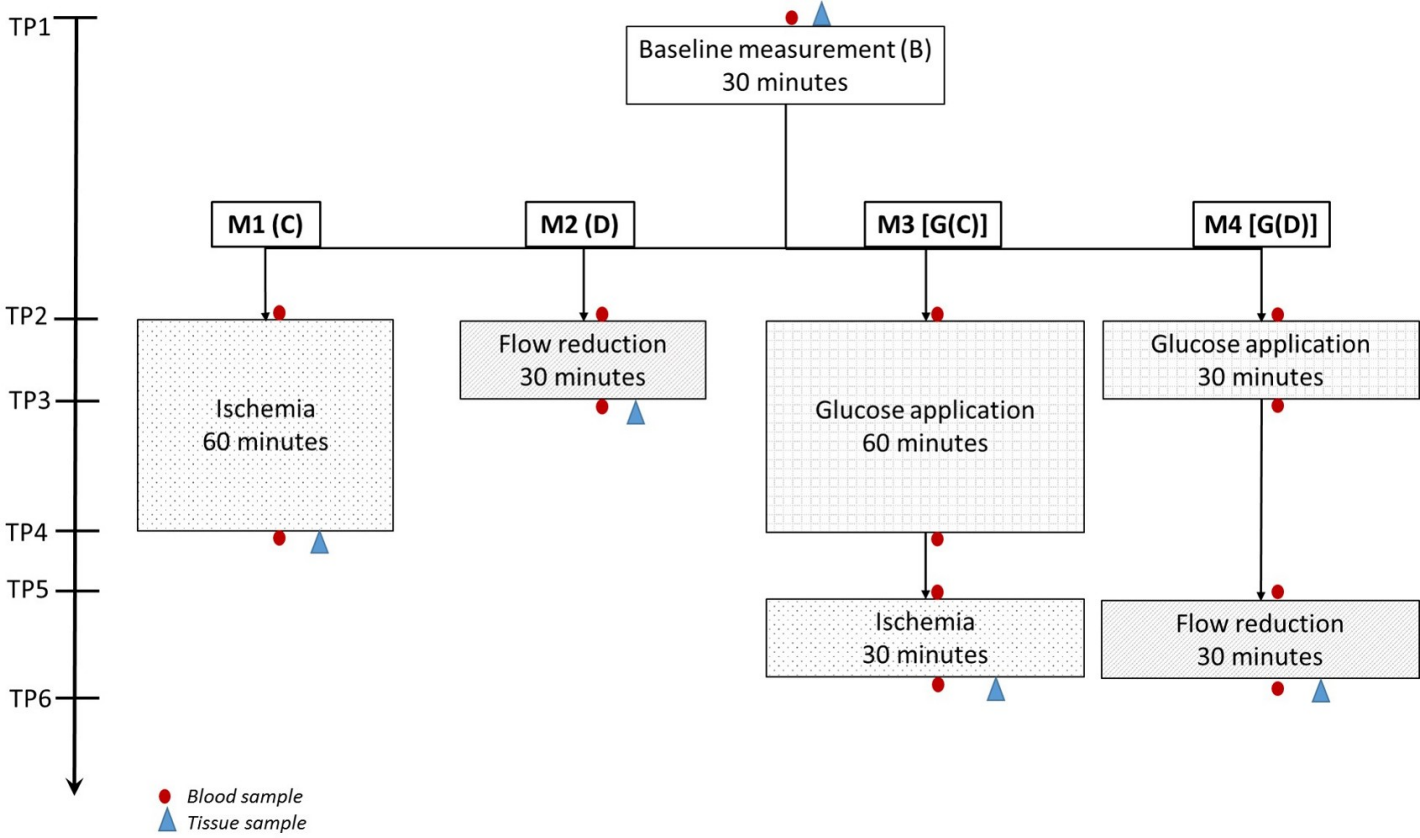
Experimental procedure showing the four groups (methods 1–4) and the time points for blood and tissue sampling.

The experimental procedure is outlined in Fig 5. In all four experimental groups (method 1–4) the main procedure was preceded by a 30 minute baseline measurement (treatment B). The main procedure differed according to the method: method 1 –clamping (treatment C), i.e. ischemia induced by ligation of the feeding vessels; method 2 –flow reduction (treatment D), i.e. reduction of blood flow to approximately 50% of the baseline blood flow measured with the outer O2C probe and induced by compression of the feeding vessels; method 3 –intraluminal application of 20 ml glucose 40% (treatment G) with subsequent ischemia; method 4—application of 20 ml glucose 40% (treatment G) with subsequent flow reduction. After termination of the measurements in methods 2 and 4 the feeding vessels of the respective intestinal loops were ligated in order to prevent systemic influence from washed in metabolites. After the end of the experiment, the animals were euthanized using pentobarbital.

### Tissue sampling and histology

Following the laparotomy and surgical preparation a biopsy was taken from the small intestine as baseline sample. At the end of the respective main procedure a biopsy was taken from each intestinal loop (Fig 5). After fixation in a PBS buffered 10% formalin solution the tissue samples were embedded in paraffin. 5 μm thick sections were cut and a hematoxylin and eosin stain (H&E stain) was performed according to our standard laboratory protocol. The stained sections were analyzed by a blinded pathologist and graded for morphological changes according to the scoring system proposed by Hierholzer et al. [11]: Grade 0—no specific pathologic changes; Grade 1—mild mucosal damage; Grade 2—moderate damage; Grade 3—extensive damage; Grade 4—severe damage and necrosis.

### Blood sampling

As shown in Fig 5, arterial and venous blood gas analysis samples were gathered at six time points (T1-T6) starting at the beginning of the baseline measurement (T1). Further samples were taken at the beginning and at the end of each treatment (T2-T6). The blood gas samples were analyzed using a Radiometer ABL 615 blood gas analyzer (Copenhagen, Denmark). The oxygen saturation, pH, glucose and lactate levels as well as arteriovenous differences in pO2 and pCO2 were measured.

### O2C monitoring

As reference method for the assessment of the local microcirculation in the intestinal mucosa and serosa we used “O2C” monitoring (oxygen to see, LEA Medizintechnik GmbH, Giessen, Germany). O2C uses a combined technology of laser doppler measurements to determine microcirculatory blood flow and velocity and tissue photo spectrometry for the assessment of the venular oxygen saturation (SO2) and the regional amount of haemoglobin (rHb). The mucosal probe (LFX-8) was placed inside the intestinal lumen, while the outer probe (LFX-26) was attached to the outer intestinal surface with two sutures (Prolene 6–0). All parameters (sO2, rHb, blood velocity and blood flow) were recorded continuously for each loop.

### NMR-MOUSE measurement

A Fourier-MOUSE was used for measurement of proton self-diffusion in the intestinal tissue. The build-up and measurement technique have been described in detail before^7^. Briefly, it consists of 2 permanent magnets connected to an iron yoke and 8 additional small movable magnets, the so called shim unit. Thus, the gradient of the magnetic field is reduced by one order of magnitude and the uniformity is increased. Using one single excitation multi-echo scan and a Fourier transformation of the echo shape, the relaxation information from the entire sensitive volume (1.5 x 1.5 mm, penetration depth of 2.5mm) can be obtained with high resolution (50μm). Thus, the measurement time is reduced drastically compared to the standard Profile NMR-MOUSE. The 1.5 x 1.5 mm coil parallel to the sensor surface generates a frequency of 9.03 MHz and is used to excite ^1^H nuclei. The strength of the magnetic field B_0_ is at 0.21 T with a gradient strength G of 1.87 T/m (80 kHz/mm). The herein reported experiments were performed using a Magritek Kea spectrometer.

#### Measuring the diffusion coefficient

Self-diffusion was measured with the steady gradient stimulated echo pulse sequence (SGSTE) [12]. By varying the time constant τ_1_ from measurement to measurement in a given range, the diffusion coefficient can be calculated using the slope of a linear regression of the normalized intensity I/I_0_.

$$\ln\left(\frac{I}{I_{0}}\right)=-\gamma^{2}G^{2}\tau_{1}^{2}(\tau_{2}+\frac{2}{3}\tau_{1})D^{12}$$

The gradient G_0_ being given by the magnet geometry, the parameters τ_1_ and τ_2_ need to be chosen suitably to mark initial and final positions (τ_1_) and to diffuse from the initial to the final position (τ_2_). Each diffusion measurement in this project was performed using the same parameters to assure comparability of the results. The parameters were optimized in order to resolve two different diffusion coefficients which can be extracted by two linear fits and are here called the fast (FC) and the slow component (SC). The parameters used in this study are shown in Table 1.

**Table 1:** Measurement parameters used in diffusion measurements with the Fourier-MOUSE.

Parameter	Value	Parameter	Value
Frequency	9.03 MHz	Acquisition time	0.227 ms
No.^a^ Echoes	256	Echo time	300 μs
Pulse length	5.5 ms	Gradient G	1.8 Tm-1
90° pulse	-12 dB	180°pulse	-6 dB
τ_1_ min. ^b^	0.05 ms	τ2	5 ms
No. ^a^ Increments	10	D (estimated)	0.5 10–9 m^2^s-1
No. ^a^ Scans	16	Receiver Gain	31
Repetition Time	1000 ms		

^a^ No.: number

^b^ Min: minutes

### Statistical analysis

Continuous variables are expressed as mean values ± standard deviation. Linear mixed models with repeated measures were chosen in order to investigate the O2C data of the landrace pigs [13]. 4 methods were compared by applying 4 different treatment sequences to different slings within the pigs. We evaluated the 4 outcome parameters ‘Flow’, ‘rHb’, ‘Velocity’ and ‘sO2’ separately and distinguished between inner and outer probe measurements. The difference of the outcome at time t to baseline was modelled as dependent variable. For each outcome and probe type, we included a continuous fixed time effect, a treatment effect (3-levels), a method effect (4 levels) and a treatment by method interaction effect. Each outcome was corrected for the baseline value (fixed covariate). Additionally, a random sling effect nested in the animal effect was modeled [14]. We assumed that the measurements at the different slings within the pigs were independent; corresponding to the independence of the methods.

The BGA data were evaluated for the 8 outcomes ‘glucose’, ‘HB’, ‘Hkt’, ‘Lac’, ‘pCO2’, ‘sO2’, ‘P02’, and ‘Ph’. For the linear models, a fixed (categorical) time effect (5-levels), a method effect (4 levels) and the baseline outcome measurement (covariate) were considered. Similarly to the O2C model, a random sling effect nested in a pig effect was modeled together with a random pig effect.

For the MOUSE data linear mixed models with repeated measures were fitted for the difference in the fast and in the slow component to baseline, including a fixed time effect (9 levels, repeated factor), a fixed method effect (5 levels) and the baseline outcome measurement as fixed covariate. To account for dependencies, a random pig effect was included. A random sling effect was not estimable, and thus had to be neglected. Observations in different methods were treated as independent and evaluated on pig level.

Within all linear models, interaction terms with time were investigated. However, they were only included if there were significant. In case of the NMR-MOUSE data interaction effects were not estimable. For all repeated-measure analyses a Kenward Rodgers adjustment was chosen due to the small sample size. The requirements of normally distributed residuals and homoscedasticity were checked using normal probability and residual plots. Post-hoc tests were conducted to compare endpoints between factor expressions/groups. For post-hoc analysis, the p-values were adjusted using the Tukey-Kramer procedure. The outcome parameters PO2 and Ph of the BGA analysis were logarithmized to meet the requirements.

For the histological analysis, the Hierholzer score was investigated for each sling in dependence of the method and the time effect separately via a Fisher exact test.

We assessed any effect in the final multivariable models as significant, if the corresponding P value fell below the 5% margin. All analyses were performed in an explorative manner. Mean plots with SEM bars and boxplots were chosen to present the distribution of selected factors over time grouped by treatment method.

Statistical analysis was performed using SAS for Windows, Version 9.4 (SAS Institute, Cary, NC, USA), ‘Proc Mixed’ resp. ‘proc HPMixed’ was used for the repeated measure analysis.

## Results

### Blood gas analysis

There was no significant overall time or method effect for the parameters glucose (p_time_ = 0.4648, p_method_ = 0.2696), hemoglobin concentration (p_time_ = 0.9629, p_method_ = 0.7173), hematocrit (p_time_ = 0.9846, p_method_ = 0.6814), sO2 (p_time_ = 0.8153, p_method_ = 0.7835) and pH (p_time_ = 0.0886, p_method_ = 0.7206).

**Fig 6:**
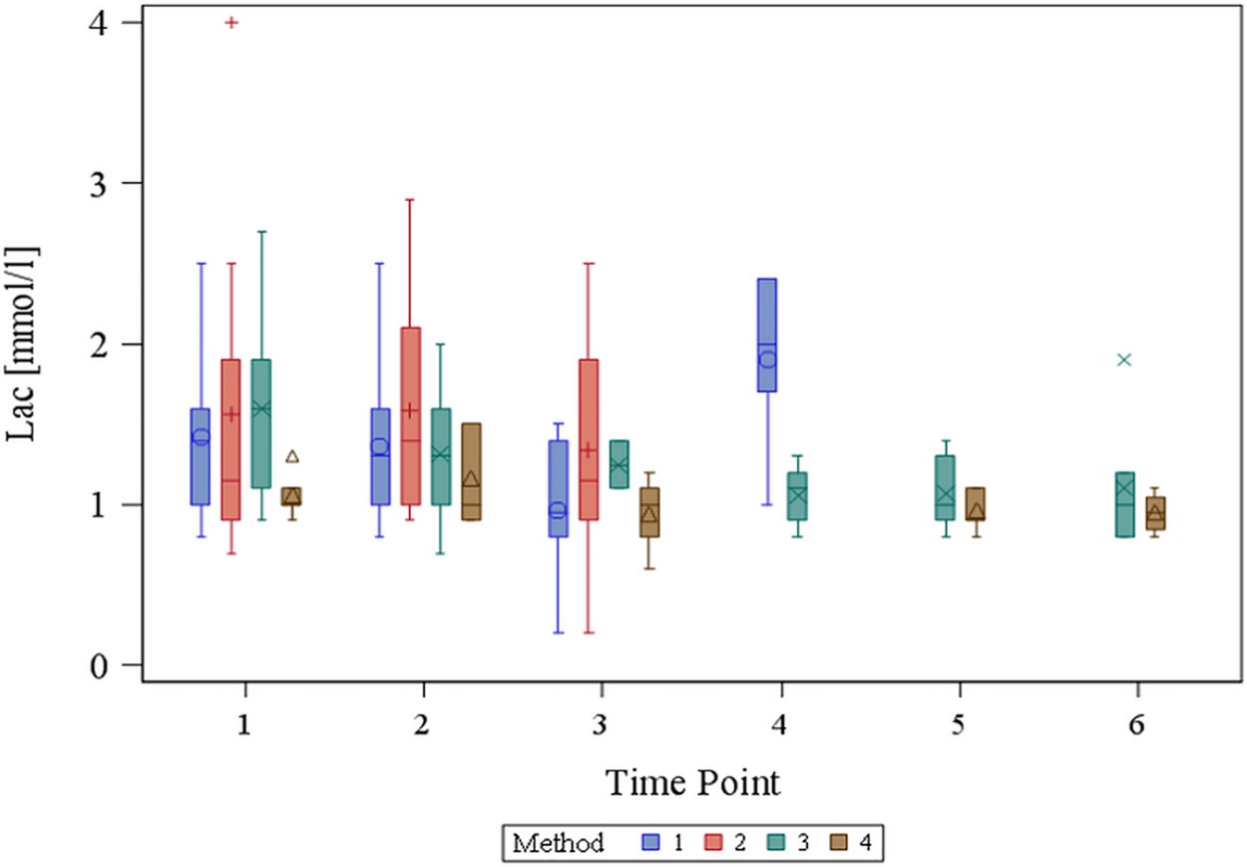
Box plot for the parameter lactate concentration. There was a significant overall time effect (p_time_ = 0.0161) with a statistically significant difference between TP2 and TP6 (adjusted p-value: p _(TP2 vs. TP6)_ = 0.0369).

For the parameter lactate concentration, the overall method effect was not significant (p_method_ = 0.1036), but there was a significant overall time effect (p_time_ = 0.0161) with a statistically significant difference between TP2 and TP6 (adjusted p-value: p _(TP2 vs. TP6)_ = 0.0369). Fig 6 shows the respective box plot. For pO2, there was a significance for the overall method effect (p_method_ = 0.0464), but the post-hoc tests showed no significant differences in the adjusted p-values (p _(M2 vs. M1)_ = 0.9494, p _(M3 vs. M1)_ = 0.9494, p _(M4 vs. M1)_ = 0.0637, p _(M2 vs. M3)_ = 0.2744, p _(M2 vs. M4)_ = 0.1704). The overall time effect for pO2 was not significant (p_time_ = 0.1217). The respective box-plot is shown in S1 Fig.

### O2C

#### SO2 –inner probe

Measurements of the inner probe showed a significant reduction of SO2 in comparison to the baseline value during both clamping and flow reduction with or without prior glucose application (p _Clamping (method 1)_ < .0001, p _Clamping (method 3)_ < .0001, p _flow reduction (method 2)_ < .0001, p _flow reduction (method 4)_ < .0001). There was no significant difference from the baseline value during glucose application (p _glucose (method 3)_ = 0.5420, p _glucose (method 4)_ = 0.4505). The results are shown in S2 Fig.

#### SO2 –outer probe

The measurements of the outer probe showed similar results to those of the inner probe with a significant reduction of sO2 during clamping with or without prior glucose application as well as during flow reduction in method 2 (p _Clamping (method 1)_ < .0001, p _Clamping (method 3)_ < .0001, p _flow reduction (method 2)_ < .0001). However, there was no significant reduction of SO2 during flow reduction in method 4 (p _flow reduction (method 4)_ = 0.2351). There was no significant change of SO2 in the outer probe measurement during glucose application (p _glucose (method 3)_ = 0.9809, p _glucose (method 4)_ = 0.7158). The results are shown in S3 Fig.

#### rHb–inner probe

In the inner probe, rHb was significantly increased in comparison to the baseline value during clamping in methods 1 and 3 as well as during flow reduction in method 2 (p _Clamping (method 1)_ = 0.0007, p _Clamping (method 3)_ = 0.0119, p _flow reduction (method 2)_ = 0.0043). There was no significant difference from the baseline during glucose application (p _glucose (method 3)_ = 0.2213, p _glucose (method 4)_ = 0.6423) and flow reduction after prior glucose application (p _flow reduction (method 4)_ = 0.2374). The results are shown in S4 Fig.

#### rHb–outer probe

Measurements of rHb in the outer probe showed a statistically significant decrease in comparison to the baseline during clamping in methods 1 and 3 (p _Clamping (method 1)_ = 0.0119, p _Clamping (method 3)_ = 0.0078) as well as during glucose application (p _glucose (method 3)_ = 0.0141, p _glucose (method 4)_ = 0.0438). There was no significant difference from the baseline during flow reduction in methods 2 and 4 (p _flow reduction (method 2)_ = 0.7198, p _flow reduction (method 4)_ = 0.2838). The results are shown in S5 Fig.

#### Flow–inner probe

The microcirculatory flow measurement of the inner probe showed a significant decrease compared to the baseline value during clamping and flow reduction both with and without prior glucose application (p _Clamping (method 1)_ < .0001, p _Clamping (method 3)_ = 0.0007, p _flow reduction (method 2)_ < .0001, p _flow reduction (method 4)_ < .0001). There was no significant difference from the baseline during glucose application (p _glucose (method 3)_ = 0.1129, p _glucose (method 4)_ = 0.8781). The reduction of microcirculatory flow during clamping was significantly smaller after prior glucose application (p _clamping (method 3) vs. clamping (method 1)_ = 0.0039). The results are shown in S6 Fig.

#### Flow–outer probe

The results of microcirculatory flow measurement in the outer probe showed a significant reduction from baseline value during clamping in methods 1 and 3 as well as during flow reduction in methods 2 and 4 (p _Clamping (method 1)_ = 0.0002, p _Clamping (method 3)_ < .0001, p _flow reduction (method 2)_ = 0.0008, p _flow reduction (method 4)_ = 0.0183). The results are shown in S7 Fig.

#### Velocity–inner probe

In the inner probe measurement, microcirculatory blood flow velocity was significantly decreased from baseline value during clamping with and without prior glucose application (p _Clamping (method 1)_ < .0001, p _Clamping (method 3)_ < .0001), during flow reduction with and without prior glucose application (p _flow reduction (method 2)_ < .0001, p _flow reduction (method 4)_ < .0001) and during glucose application in method 4 (p _glucose (method 4)_ < .0001). There was no statistically significant difference from baseline value during glucose application in method 3 (p _glucose (method 3)_ = 0.9592). The results are shown in S8 Fig.

#### Velocity–outer probe

Microcirculatory blood flow velocity in the outer probe measurement was significantly decreased from baseline value during clamping with and without prior glucose application (p _Clamping (method 1)_ < .0001, p _Clamping (method 3)_ = 0.0007), during flow reduction without prior glucose application (p _flow reduction (method 2)_ = 0.0183) and during glucose application in method 3 (p _glucose (method 3)_ = 0.0033). There was no statistically significant difference from baseline value during flow reduction with prior glucose application (p _flow reduction (method 4)_ = 0.0810) and during glucose application in method 4 (p _glucose (method 4)_ = 0.7718). The results are shown in S9 Fig.

### NMR-MOUSE

**Fig 7:**
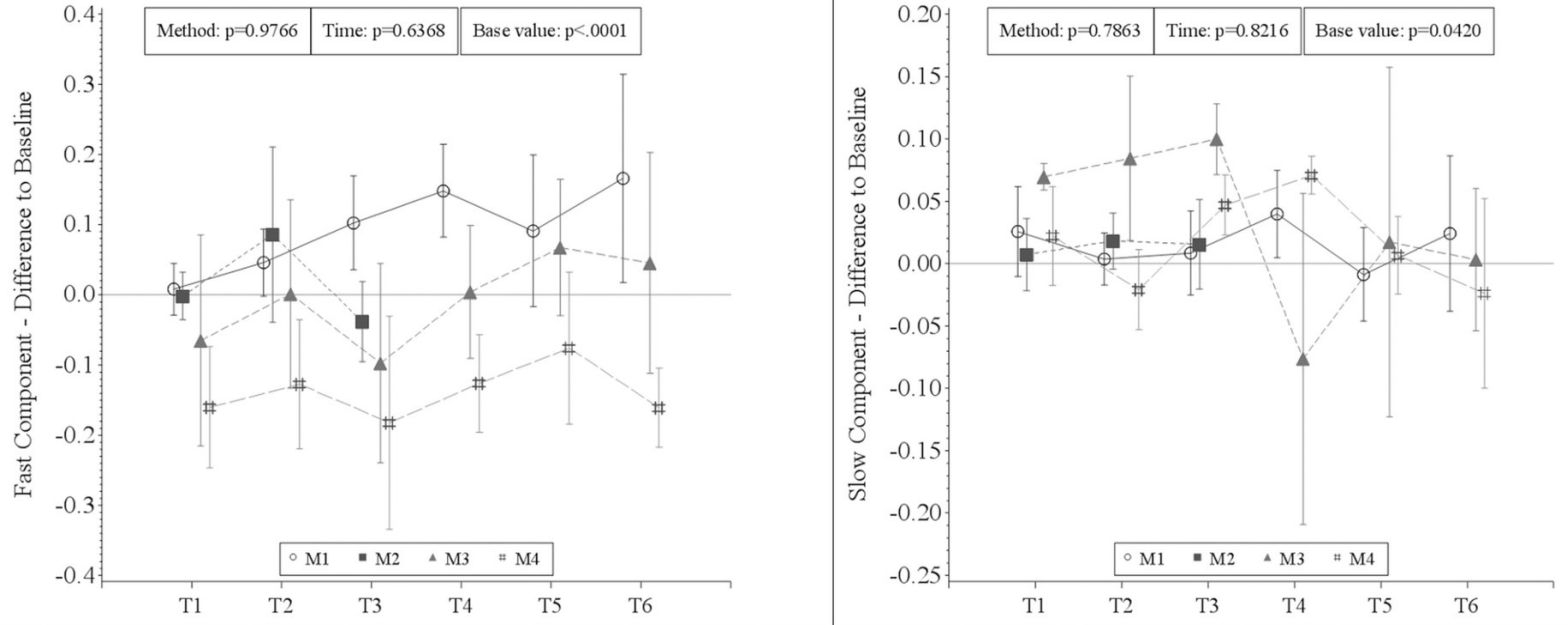
Results of the NMR-MOUSE measurement. There was no statistical significance but a trend to increase during ischemia (M1) and to decrease during flow reduction (M2) in the fast component.

There was no statistically significant overall time or overall method effect in the fast and slow components (FC: p_time_ = 0.6368, p_method_ = 0.9766, SC: p_time_ = 0.8216, p_method_ = 0.7863). However, as shown in Fig 7, the FC tends to increase during clamping in method 1. On the other hand, it shows a trend to decrease during flow reduction in method 2.

**Fig 8:**
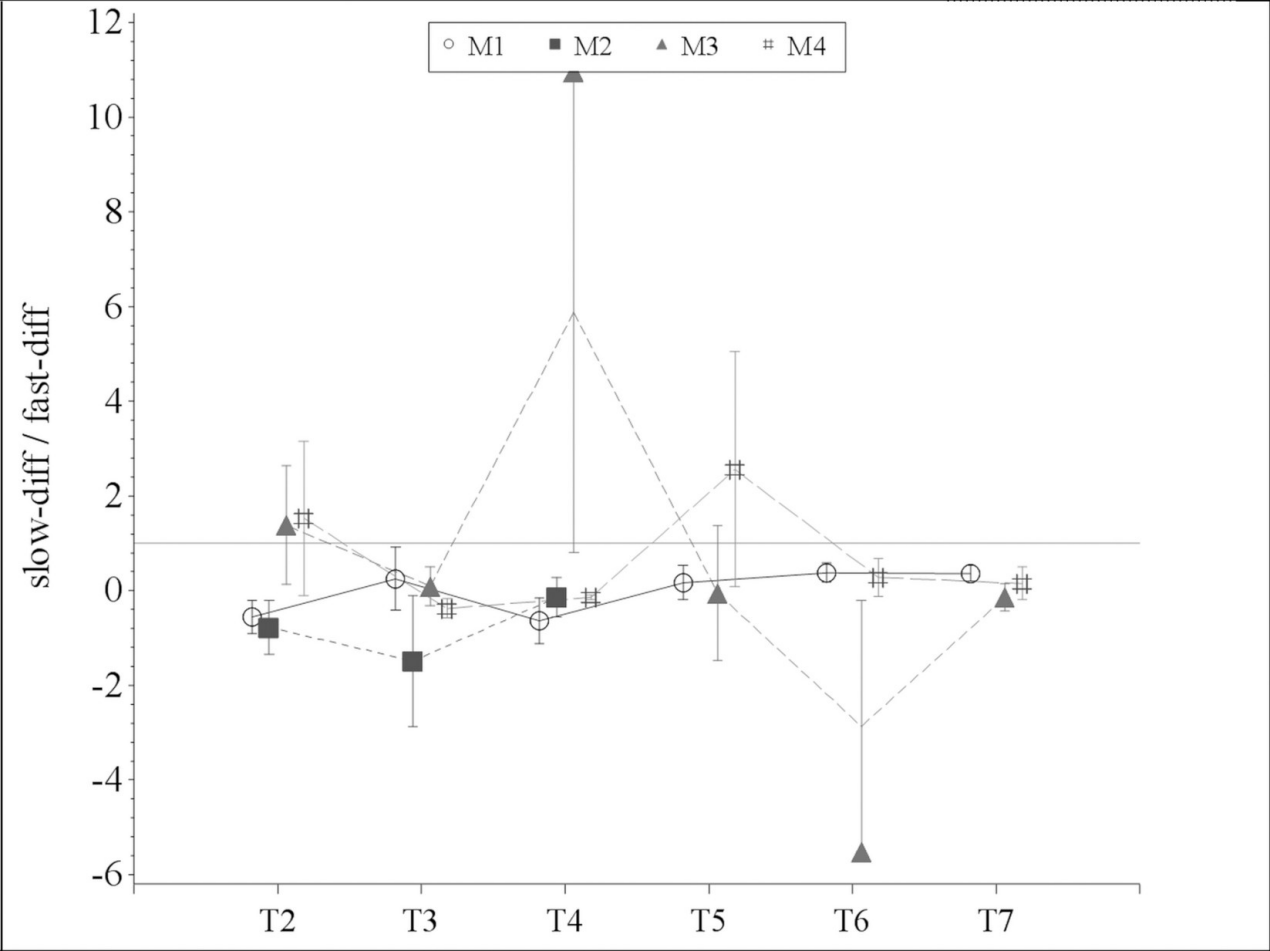
Slow-to-fast diffusion ratio of the NMR-MOUSE measurement. There was a slight shift towards slow diffusion during the main procedures in method 2 (flow reduction). At the end of glucose application in method 3 and just after the beginning of flow reduction in method 4 there was a shift towards fast diffusion with a subsequent reversal towards slow diffusion.

The analysis of the slow-to-fast diffusion ratio showed a slight shift towards slow diffusion during the main procedures in method 2 (flow reduction). At the end of glucose application in method 3 and just after the beginning of flow reduction in method 4 there was a shift towards fast diffusion with a subsequent reversal towards slow diffusion (Fig 8).

### Histology

There was a significant association between the Hierholzer score and the method (p < .0001, Fisher Test) as well as the time points (p < .0001, Fisher-test). Higher scores were observed at later time points as well as in methods 3 and 4. Table 2 shows the respective contingency tables for time effect (A) and method effect (B).

**Table 2:** Contingency tables of the histopathological score analysis (Hierholzer score).

**A: Method effect**					
Method	Score				
	0	1	2	3	Sum
Baseline	8	0	0	0	8
Method 1—clamping	1	8	3	0	12
Method 2 – flow reduction	0	6	3	0	9
Method 3 – glucose + clamping	1	1	4	1	7
Method 4 – glucose + flow reduction	1	1	3	0	5
Sum	11	16	13	1	41
**B: Time effect**					
Time point	Score				
	0	1	2	3	Sum
1	8	0	0	0	8
3	0	13	3	0	16
4	1	1	3	0	5
6	2	2	7	1	12
Sum	11	16	13	1	41

## Discussion

The present study was the first to evaluate diffusion of the gut wall with the NMR-MOUSE. Our results show that the NMR-MOUSE is able to detect changes of the gut wall diffusion during different intestinal perfusion states although the results were not statistically significant. Even though this technology seems promising, there are still relevant technical limitations that require refinement.

Intestinal hypoperfusion/ischemia and subsequent reperfusion play an important role in the development of postoperative multi-organ dysfunction and SIRS following cardiovascular surgery with ECC [1]. ECC not only affects the intestinal microcirculation [5], but it also impairs intestinal barrier function and mucosal transport capacity after surgery [10]. Regarding the assessment of intestinal microcirculation, different preclinical imaging techniques, e.g. laser doppler perfusion imaging [15], in vivo microscopy [16], laser speckle contrast imaging [17] and fluorescence based enhanced reality [18], have been employed in experimental models of intestinal ischemia/reperfusion (I/R) injury. In view of the above-mentioned functional effects of ECC-related intestinal injury however, the present study aimed beyond the detection of microcirculatory perfusion changes, at the assessment of early functional consequences of different intestinal perfusion states by means of diffusion monitoring. The stable results of the BGA monitoring in this study allowed to exclude a systemic effect of the experimental procedures as a potential source of bias. The results of the O2C measurements confirmed the expected microcirculatory alterations [19] with reduction of SO2, microcirculatory blood flow and velocity during ischemia and flow reduction. The rHb measurement, expressing the amount of blood in the local microcirculatory vascular bed, showed a mucosal increase but a serosal decrease during ischemia. This is in accordance with previous findings of a microcirculatory perfusion shift between the gut wall layers during a suboptimal intestinal perfusion state during ECC [5].

In agreement with other experimental studies demonstrating changes in bioimpedence measurements during early small intestinal ischemia as well as I/R induced alterations of ion transport across colonic epithelium [20, 21], this study showed diffusion changes during intestinal hypoperfusion and ischemia using NMR-MOUSE monitoring. Although the herein reported results of the diffusion measurement did not show statistical significance, the opposite trends seen in the FC during clamping versus flow reduction seem to reflect the perfusion changes. The literature regarding the interpretation of the two diffusion components is controversial, but the fast diffusion component can be understood to refer to the water in the intracellular compartment whereas the slow diffusion component refers to water in the extracellular or interstitial compartment [22]. However, it has been questioned whether the two compartments can be assessed separately in vivo [23]. As to the FC in the present study, its tendency to decrease during flow reduction could be explained by a reduced cellular metabolic activity due to the lack of available energy. Consequently, all ATP-dependent transport processes are reduced, such as the sodium-potassium adenosine triphosphatase (Na^+^/K^+^-ATPase) which plays a fundamental role in cellular ion and water homeostasis and in intestinal absorption [23, 24]. The increase of the FC during ischemia on the other hand may be explained by cellular swelling and death as well as by the disruption of paracellular barriers, i.e. tight junctions, during ischemia [25, 26] causing uncontrolled and therefore faster diffusion. Indeed, microvascular leakage has been demonstrated even after short periods (30 min) of suboptimal intestinal perfusion during cardiopulmonary bypass [19]. In contrast to the FC of the diffusion measurement in the present study, the SC did not show the above-mentioned changes during different perfusion states. This may be due to the higher susceptibility of this parameter to external “noise” which reduces the precision of the measurement.

Enteral glucose administration has been shown to protect from intestinal I/R injury by reducing cell death and intestinal permeability [27, 28]. Moreover, it has been demonstrated that intraluminal glucose increases intestinal mucosal blood flow during I/R states thereby mitigating the injury [29]. In the present study however, we did not observe statistically significant alterations in the microcirculation during glucose application. Nonetheless, the microcirculatory flow reduction during hypoperfusion was attenuated after glucose administration supporting the aforementioned data on its protective effect. Despite the fact that the analysis of the slow-to-fast diffusion ratio showed some variation in the experimental groups including intraluminal glucose administration in comparison to those without glucose, the results of the NMR-MOUSE measurements were inconclusive in this regard.

Different factors have to be considered trying to explain the lack of statistical significance of the present results. First of all, in comparison to other reports on intestinal I/R injury [2, 19], the current study focused on changes during early hypoperfusion/ischemia and, therefore, did not include a reperfusion period. Consequently, the observed intestinal damage without reperfusion is smaller [30]. This is supported by the results of the present histopathological analysis that showed overall lesser score values than in previous porcine studies including a reperfusion period [2]. Furthermore, the NMR-MOUSE measurement in this study assessed proton diffusion. Yet, it is possible that the assessment of alterations in the diffusion of other ions is more sensitive to detect intestinal perfusion changes. For instance, it has been shown that changes in calcium (Ca^2+^) homeostasis due to early I/R injury can be detected using manganese-enhanced magnetic resonance imaging (MRI) in a rat model [31]. The investigation of Ca^2+^ however is not feasible using a low-field scanner such as the NMR-MOUSE. Another study confirmed the importance of changes in Ca^2+^ flux but also in chloride (Cl^-^) secretion in an experimental model of small intestinal hypoxia/reoxygenation [21]. Supporting the relevance of changes in intestinal Cl^-^ secretion during hypoxia, a direct interaction of hypoxia-inducible factor-1 (HIF-1) with the intestinal cystic fibrosis transmembrane conductance regulator (CFTR) has been reported [32]. The hypoxia-related changes in Cl^-^ flux in turn cause a modulation of sodium (Na^+^) and water flux across the intestine. Therefore, the investigation of Cl^-^ and/or Na^+^ diffusion instead of proton diffusion could be of interest for future experiments evaluating the NMR-MOUSE in the context of intestinal hypoperfusion.

Finally, it has to be taken into account that the animals in this study were juvenile with a healthy vascular system. Possibly, the diffusion changes are less pronounced thanks to functioning compensatory mechanisms. Further investigations on older animals are desirable in order to clarify this aspect.

### Study limitations

The limitations of this study include the small study population. It corresponds to the minimum number of animals required in order to achieve statistically significant results according to the a priori power analysis. It has to be taken into account that not all of the complex data dependencies could be modelled and estimated within this framework. Due to the small number of pigs, the comparison of the methods within the pigs may be biased as we lack observations to investigate the pig and sling effect more precisely. Yet, the longitudinal investigation increases the power of our analysis through the number of repeated measures, as they improve the variance estimation. The random allocation of the methods applied to the slings within the pigs was used to protect from selection bias a well as possible.

Additionally, we have found during first experiments preceding the herein reported ones that the current maximum penetration depth of the NMR-MOUSE (2.5mm) does not allow a completely non-invasive diffusion assessment at the level of interest, i.e. at the mucosal to submucosal transition layer, due to intestinal swelling after laparotomy and early ischemic effects [30]. Consequently, we had to adapt the experimental protocol as described to opening the intestinal loops at the antimesenteric side allowing direct mucosal measurement. The first experiments without the antimesenteric incision were excluded from the present study because of the different methodology. For future investigations, an increase of the penetration depth would be commendable in order to allow a non-invasive proceeding.

## Conclusion

Intraoperative monitoring of intestinal diffusion using the NMR-MOUSE is feasible in an experimental setting in a large animal model. The first results regarding the assessment of early effects of pathological intestinal perfusion states are promising. However, an optimization of the technique is necessary before an application for intestinal perfusion monitoring, e.g. for evaluation of different extracorporeal perfusion techniques in an experimental setting, is possible. Thus, further validation studies on a larger number of animals should be considered, starting with more investigations under physiological conditions and including measurements on older animals in order to clarify the possible influence of compensatory mechanisms at younger age suggested by the present results. To this end, the possibility of an adaptation of the currently available NMR-MOUSE for measurements in small animals should also be explored.

## Supporting information

S1 Fig Box plot for the parameter pO2 in the BGA measurement. (TIF)

S2 Fig Results of the O2C measurement (inner probe, parameter: sO2). (TIF)

S3 Fig Results of the O2C measurement (outer probe, parameter: sO2). (TIF)

S4 Fig Results of the O2C measurement (inner probe, parameter: rHb). (TIF)

S5 Fig Results of the O2C measurement (outer probe, parameter: rHb). (TIF)

S6 Fig Results of the O2C measurement (inner probe, parameter: Flow). (TIF)

S7 Fig Results of the O2C measurement (outer probe, parameter: Flow). (TIF)

S8 Fig Results of the O2C measurement (inner probe, parameter: Velocity). (TIF)

S9 Fig Results of the O2C measurement (outer probe, parameter: Velocity). (TIF)
