# Supplementary figures and images for: Evaluation of the NMR-MOUSE as a new method for continuous functional monitoring of the small intestine during different perfusion states in a porcine model
Source: PLoS One. 2018 Nov 2;13(11):e0206697. doi: 10.1371/journal.pone.0206697 (PMC6214547; doi:10.1371/journal.pone.0206697)

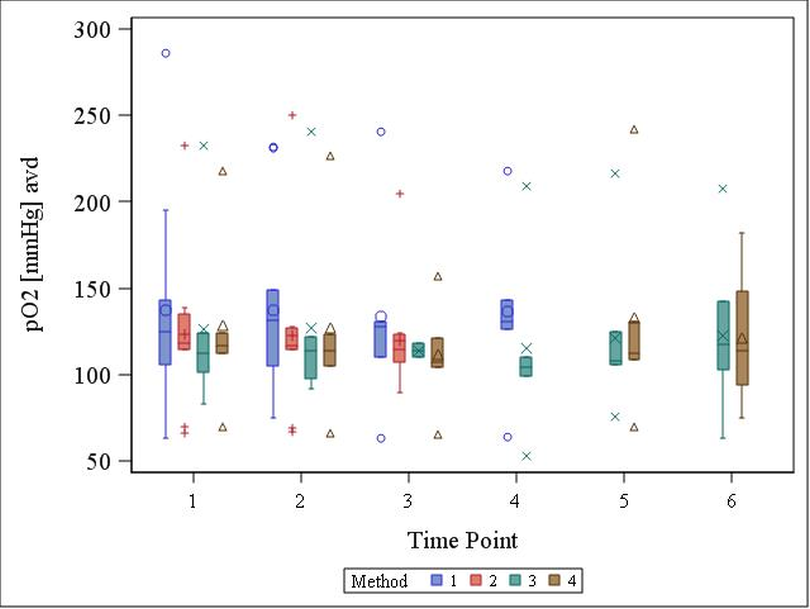

Supplement: S1 Fig — (TIF) [file pone.0206697.s001.tif]

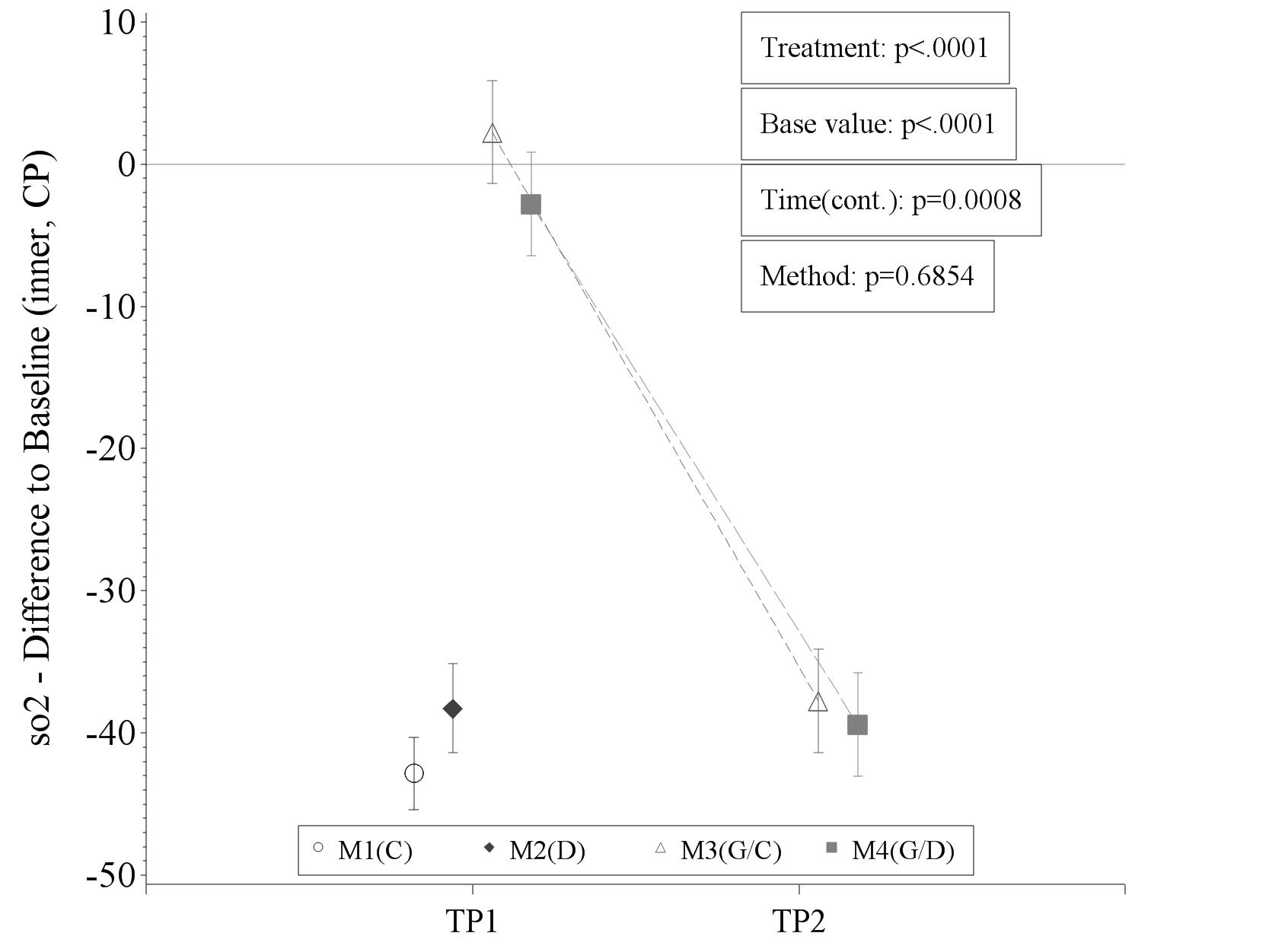

Supplement: S2 Fig — (TIF) [file pone.0206697.s002.tif]

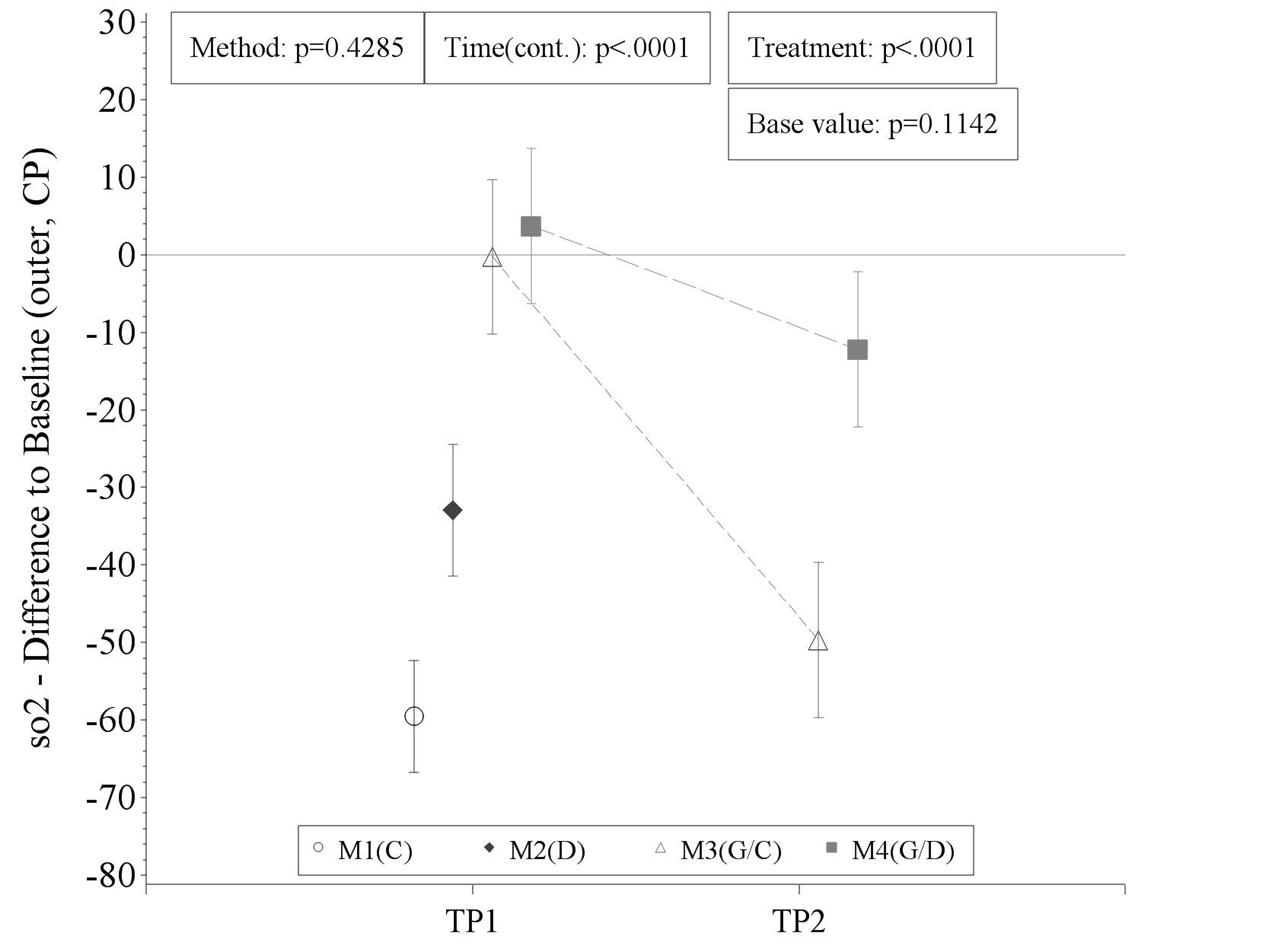

Supplement: S3 Fig — (TIF) [file pone.0206697.s003.tif]

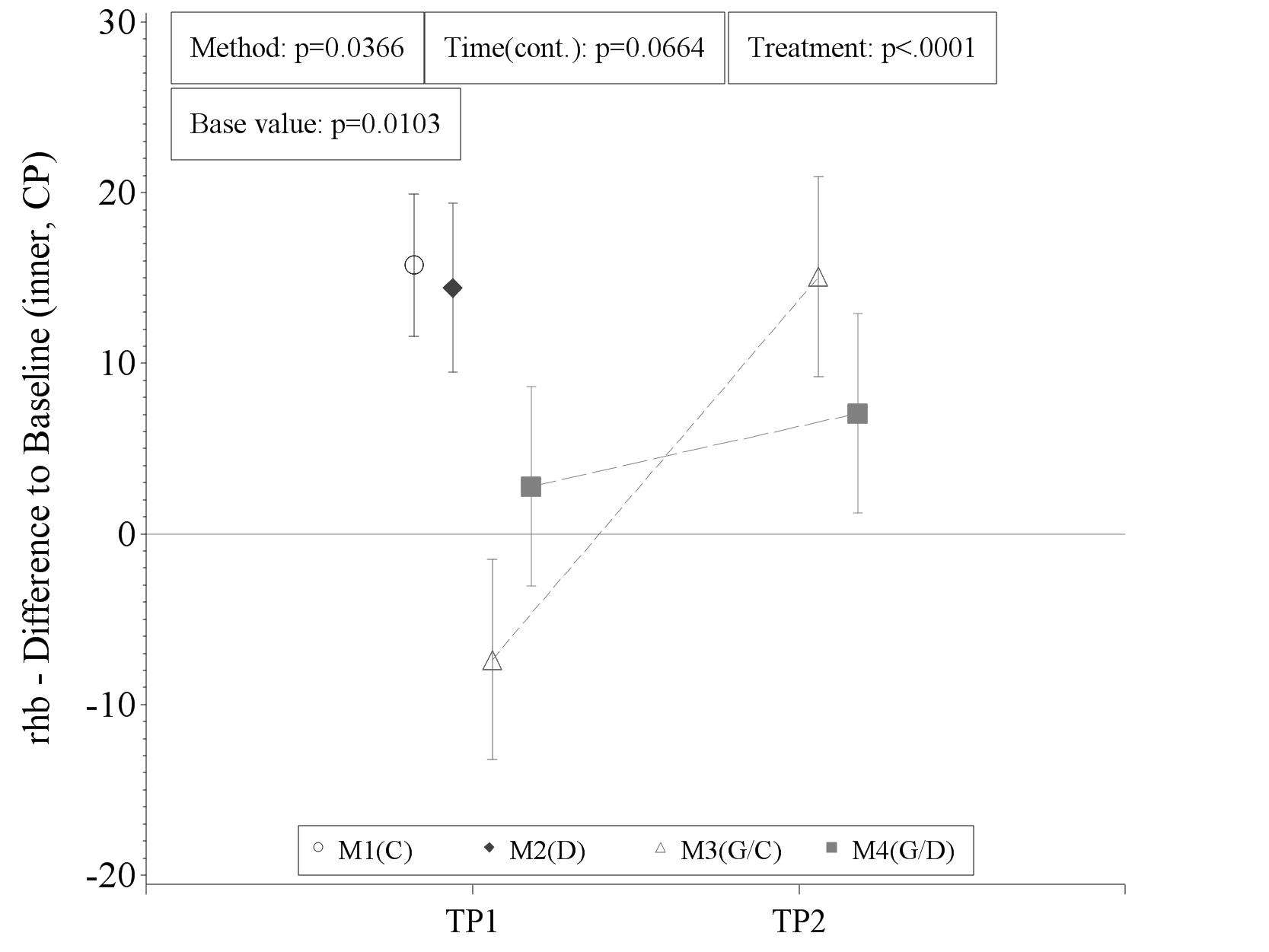

Supplement: S4 Fig — (TIF) [file pone.0206697.s004.tif]

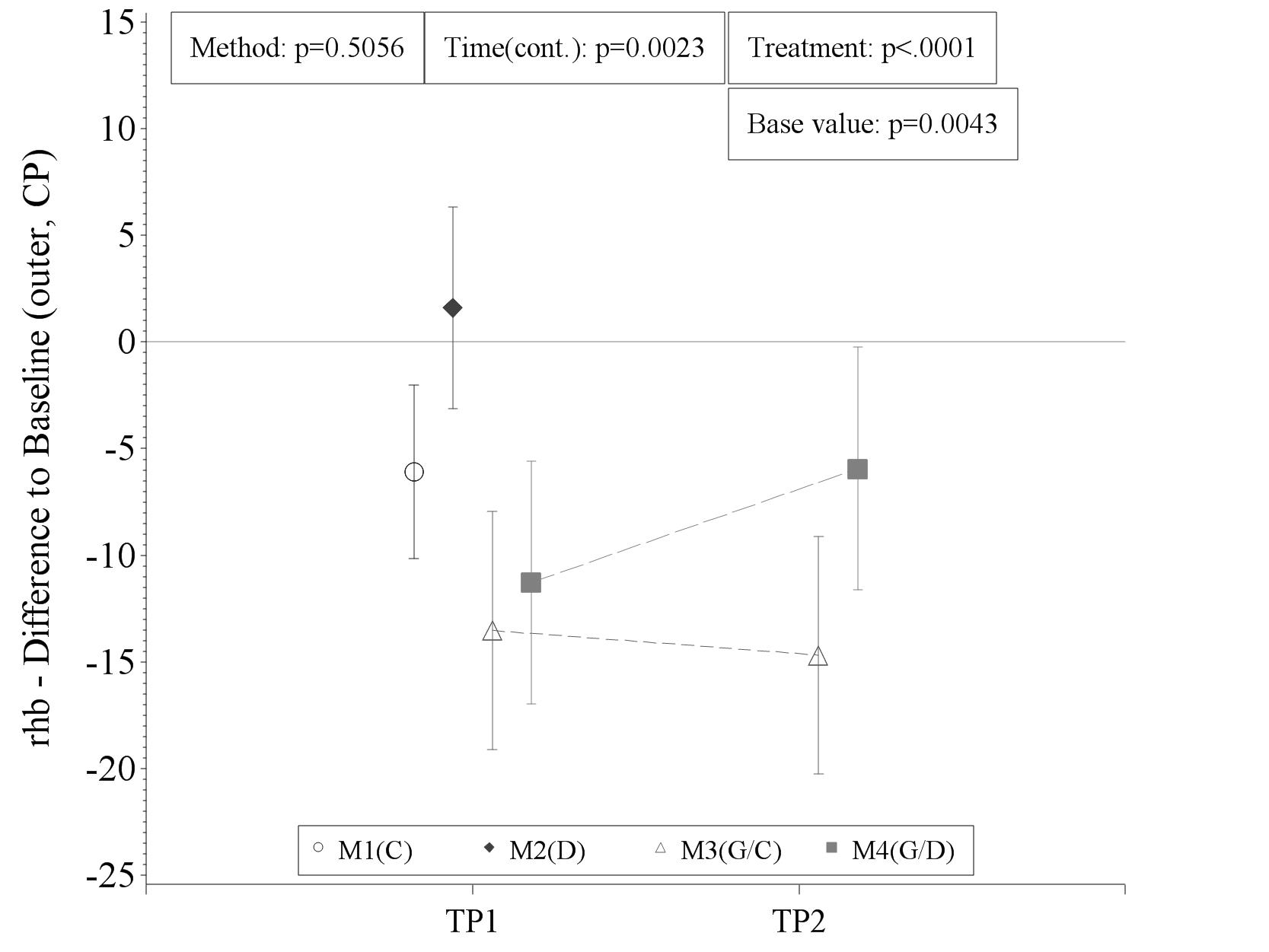

Supplement: S5 Fig — (TIF) [file pone.0206697.s005.tif]

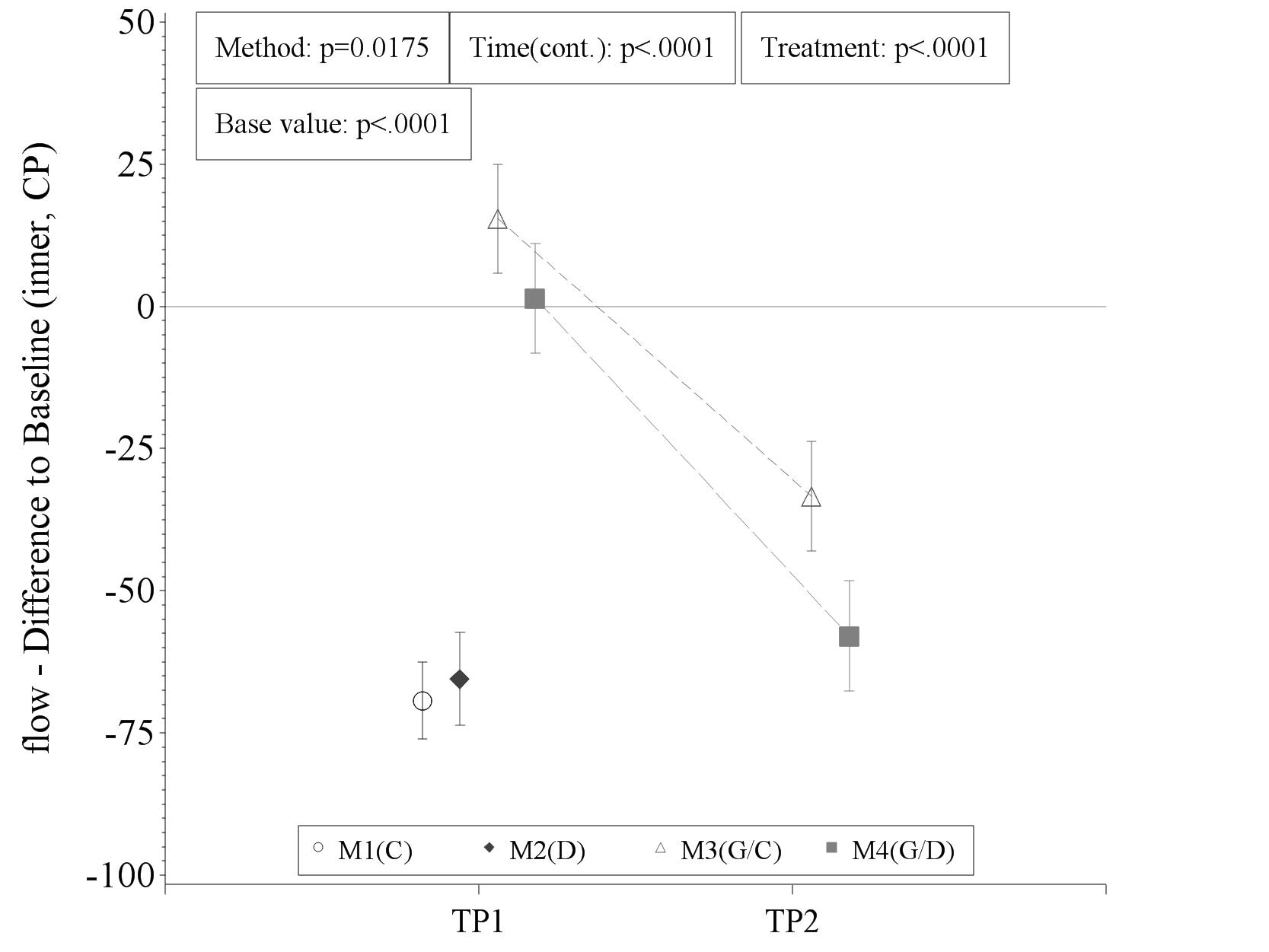

Supplement: S6 Fig — (TIF) [file pone.0206697.s006.tif]

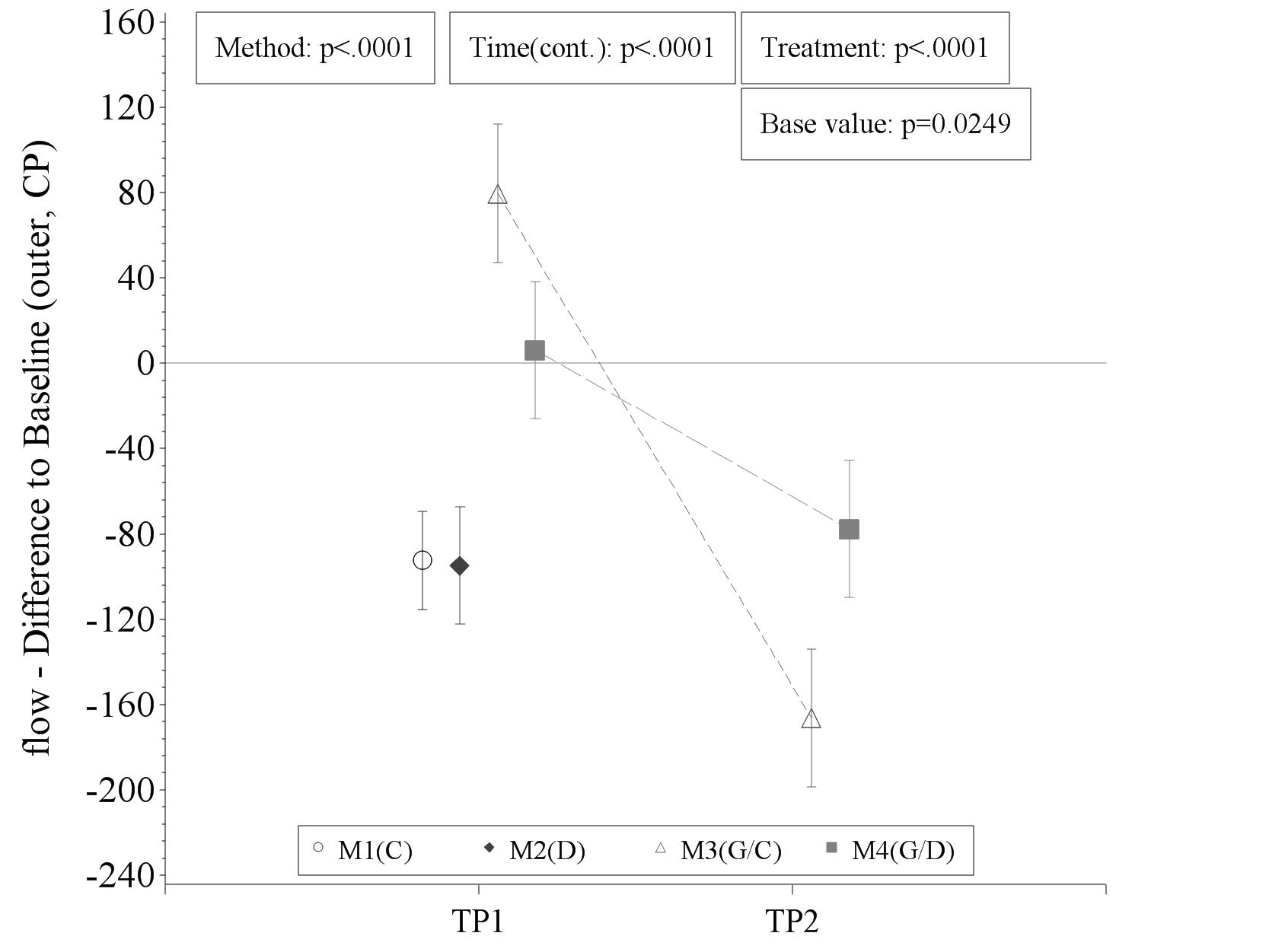

Supplement: S7 Fig — (TIF) [file pone.0206697.s007.tif]

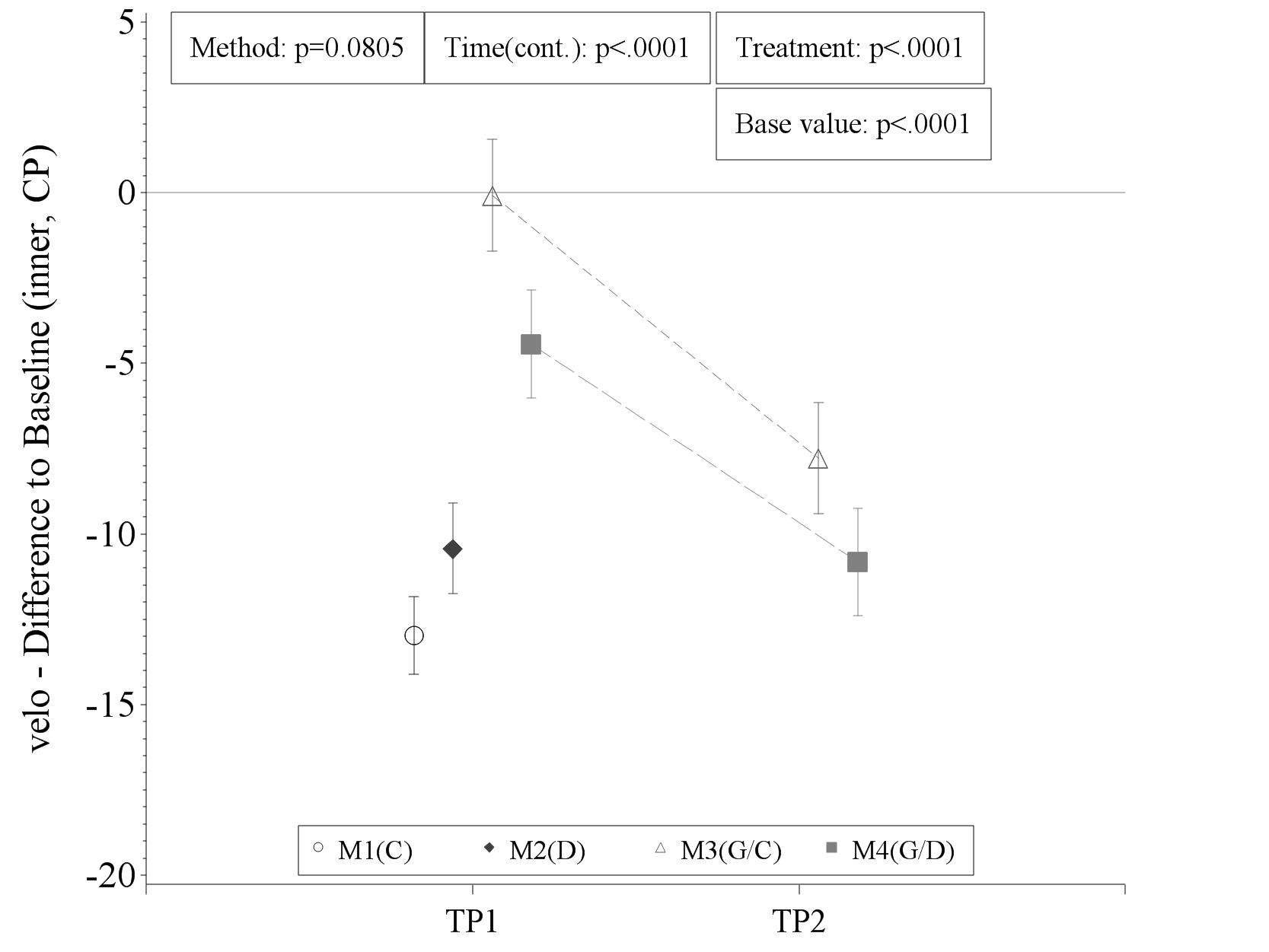

Supplement: S8 Fig — (TIF) [file pone.0206697.s008.tif]

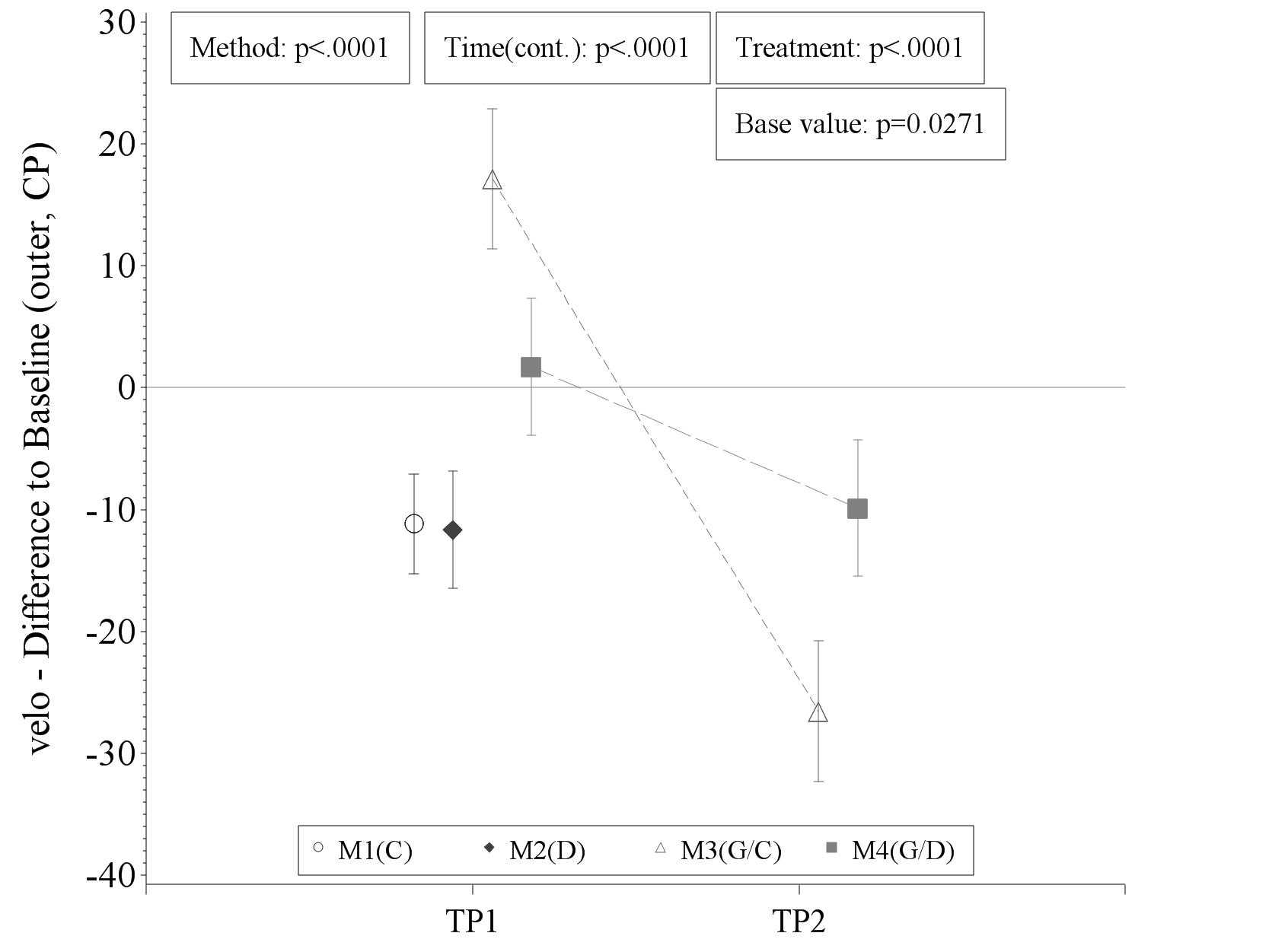

Supplement: S9 Fig — (TIF) [file pone.0206697.s009.tif]
